# Design of a Nano-Refractive Index Sensor Based on a MIM Waveguide Coupled with a Cat-Faced Resonator for Temperature Detection and Biosensing Applications

**DOI:** 10.3390/s26030826

**Published:** 2026-01-26

**Authors:** Jianhong Zheng, Shubin Yan, Chen Chen, Kecheng Ding, Yang Cui, Taiquan Wu

**Affiliations:** 1 School of Electrical and Control Engineering, North University of China, Taiyuan 030051, China; 2 School of Electrical Engineering, Zhejiang University of Water Resources and Electric Power, Hangzhou 310018, China; 3 Joint Laboratory of Intelligent Equipment and System for Water Conservancy and Hydropower Safety Monitoring of Zhejiang Province and Belarus, Hangzhou 310018, China

**Keywords:** metal–insulator–metal (MIM), SPPs, fano resonance, finite element method (FEM), nano refractive index sensor

## Abstract

This study introduces an innovative sensor architecture predicated on surface plasmon polaritons (SPPs), comprising a metal–insulator–metal (MIM) waveguide in conjunction with a cat-faced circular split resonator (TCRSW). The efficacy of the proposed nanosensor was meticulously evaluated utilizing the finite element method (FEM). It was determined that the TCRSW configuration significantly impacts the sensor’s performance. By means of a comprehensive optimization of the structural parameters, the sensor attained an apex sensitivity of 3380 nm/RIU and a figure of merit (FOM) of 56.33 in its optimal configuration. Furthermore, the study comprehensively evaluated the sensor’s applicability for temperature sensing, demonstrating a measured temperature sensitivity of 1.673 nm/°C. Meanwhile, the application of the proposed structure in biosensing was comprehensively evaluated. When employed as a concentration sensor for detecting sodium and potassium ion solutions, the maximum achievable sensitivities reached 0.49 mg·d/L and 0.6375 mg·d/L, respectively, which highlights its significant potential not only for high-precision temperature monitoring but also for sensitive and reliable biosensing applications. Additionally, the proposed nanosensor holds considerable promise for applications in other nanophotonic fields.

## 1. Introduction

Surface plasmon polaritons (SPPs) constitute electromagnetic interfacial waves propagating at a metal–dielectric boundary, emanating from the coupling between coherent oscillations of conduction electrons and the incident photon field. When incident light waves impinge upon the metallic surface, specific frequencies of these waves can excite the oscillations of the free electrons, thereby generating SPPs [1,2]. Surface Plasmon Polaritons (SPPs) are critically important because they can confine electromagnetic energy at a subwavelength scale and generate significant field enhancement. This capability enables them to surpass the resolution limits of conventional optics and underpins the field of nanophotonics [3,4].

Fano resonance epitomizes an asymmetric resonant phenomenon engendered by the quantum interference interplay between discrete and continuous states, delineated by the emergence of acute and non-symmetrical spectral lineaments within the scattering spectrum [5,6]. In photonic systems, this resonance is typically achieved by designing resonator structures; the resonator provides discrete localized states, while an adjacent waveguide or transmission channel provides a broad-spectrum continuum, and their interference via near-field coupling gives rise to Fano resonance [7,8,9]. This mechanism is particularly pronounced in MIM waveguide systems. The MIM waveguide functions as a low-loss, highly confined continuum transmission medium. When it is laterally coupled to a plasmonic resonator, the modes of the cavity (discrete states) exhibit strong interference with the continuum modes of the waveguide, culminating in the manifestation of a Fano resonance apex across the transmittance profile, distinguished by a heightened quality factor and markedly precipitous edges [10,11,12]. The FWHM of this resonance manifests an extraordinarily constricted profile, conferring apex sensitivity to fluctuations in the ambient refractive index, with a responsiveness on the magnitude of 10^3^–10^4^ nm/RIU [13]. Consequently, it finds widespread applications in optical switches [14,15], couplers [16], filters [17,18], nanosensors [19,20], and logic gates [21].

Fano resonances produced by the interaction between MIM waveguides and optical resonators have been widely studied, particularly for nanoscale sensor systems. This approach has now evolved into a core strategy for designing a range of devices, including ultrasensitive sensors, low-power photonic components, and selective filters. Zhu et al. [22] introduced an innovative Fano resonance detector characterized by a concentric split-ring resonator, characterized by a peak sensitivity of 1250 nm/RIU with a concurrent FOM of 54. In a separate study, Chen et al. [23] developed a sensor utilizing a MIM waveguide in conjunction with semi-elliptical and quadrilateral ring resonators; the transmittance profile of this configuration exhibited a pronounced Fano resonance profile, characterized by a peak sensitivity of 1384 nm/RIU with a concurrent FOM of 28.4. Furthermore, Chou et al. [24] performed an extensive computational and theoretical analysis of a plasmonic refractive index sensor, which comprised an MIM waveguide side-coupled to a nanoring embedded with a silver nanorod, with documented performance metrics of 2080 nm/RIU for peak sensitivity and 29.67 for FOM. In addition, Gao et al. designed a metal–insulator–metal (MIM) waveguide structure with a baffle, which couples a square resonant cavity and a silver elliptical cylinder. The transmission spectrum of this structure exhibits multiple Fano resonances, with the characteristic peak sensitivity reaching up to 1942 nm/RIU while achieving a quality factor (FOM) of up to 1.1 × 10^5^ [25]. Meanwhile, Gu et al. designed a hexagonal non-closely packed thin silver nanoshell array. This structure supports high Q value strong lattice plasma resonance, characterized by a Q factor as high as 610 (resonance wavelength 870 nm) [26]. In addition, Peng et al. optimized and designed a nano-metal grating sensor based on surface plasmon resonance through the finite-difference time-domain method. This sensor is suitable for protein detection and flexible stress–strain and other multimodal forms of sensing. Its characteristic is that the sensitivity can reach 0.417 μm/RIU under the optimal parameters [27]. In these investigations, sensitivity and FOM have emerged as pivotal factors constraining the advancement of refractive index sensors. However, achieving a simultaneous balance between high sensitivity and a high FOM remains a pivotal challenge.

This study presents a novel nanoscale sensor that addresses this challenge through a unique geometric innovation. The key innovation of the proposed “cat-face” resonator (tomato-faced circular split ring resonator, TCRSW) lies in its multi-embedded and hierarchical design, engineered to create a synergistic coupling scheme between multiple spatially distributed discrete resonant modes and the broadband continuum of the waveguide. Compared to conventional single- or dual-cavity designs, this architecture enables significantly stronger magnetic field concentration and mode interaction, which directly leads to a sharper and more asymmetric Fano resonance spectral line shape. It is this tailored line shape, originating from the novel coupling scheme, that fundamentally enhances the sensing performance. The transmission characteristics were simulated using the finite element method in COMSOL Multiphysics 5.6. By examining the influence of various geometric parameters, the sensor was optimized to achieve a peak sensitivity of 3380 nm/RIU and a figure of merit (FOM) of 56.33. This work therefore provides an in-depth investigation of a new resonator design principle that effectively balances both high sensitivity and high FOM, advancing the design paradigm for Fano-resonant plasmonic sensors.

## 2. Structural Model and Research Methods

**Figure 1:**
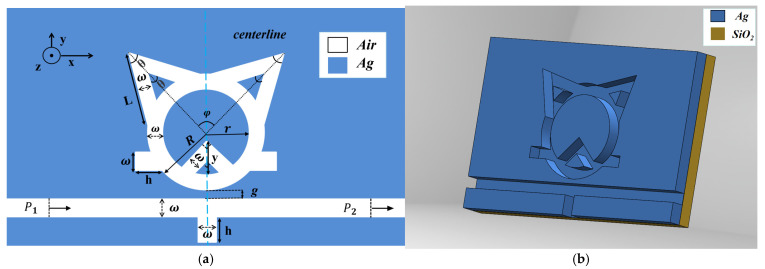
(**a**) Two-dimensional model of the nanosensor. (**b**) Three-dimensional model of the nanosensor.

The variation in the electromagnetic field across the thickness of the sensor structure is negligible, given that the incident wavelength is much larger than its characteristic thickness. Therefore, a two-dimensional model can be employed for accurate and computationally efficient simulation of such subwavelength structures, rather than a more complex three-dimensional model. The symmetric sensor structure consists of a waveguide with a rectangular stub integrated with a cat-faced circular split ring resonator (TCRSW). The designed two-dimensional and three-dimensional schematics of the sensor structure are rendered in Figure 1a and Figure 1b, respectively. The key geometric parameters are articulated as follows: R denotes the outer radius of the larger ring, r represents its inner radius, and ω indicates the width of the MIM waveguide. Additionally, the structure includes two symmetric equilateral triangular cavities, three rectangular cavities, and an isosceles right-angled triangular cavity, while h signifies the height of the three rectangular cavities. θ is the vertex angle of the symmetric equilateral triangular cavities (set to 60°), φ is the angle between the central axes of the two symmetric triangular cavities relative to the ring center, y is the leg length of the external isosceles right-angled triangle, and L represents the side length of the external equilateral triangles, while g denotes the coupling distance between the waveguide and the TCRSW structure. The width ω is conventionally established at 50 nm to enable the transmission of only the fundamental transverse magnetic mode.

In Figure 1a, the blue and white regions correspond to silver metal and the air dielectric, respectively. Figure 1b better illustrates the 3D structural features, where a quartz substrate is utilized. Silver was selected as the metallic material in this study primarily due to its exceptional electromagnetic properties and excellent chemical stability. On the one hand, silver exhibits a low imaginary part of its dielectric constant, manifesting as exceptionally low ohmic losses, which is conducive to achieving strong electromagnetic field localization and enhancement. On the other hand, the inherent high chemical stability and corrosion resistance of silver ensure the long-term reliability of the device in complex environments, which is crucial for maintaining the stability of the plasmonic resonance effects. This distinctive amalgamation of properties renders silver an optimal choice for the development of advanced plasmonic devices.

The physical basis for the operation of this sensor is Fano resonance, which stems from the constructive and destructive interference between a discrete narrowband resonant state and a continuous broadband state. Its typical optical response (transmittance T) can be described by the classic Fano Formula [28]:

(1) $$T\left(\lambda \right)=A\frac{{\left(q+\epsilon \right)}^{2}}{1+\epsilon }+B$$

Among them, $\epsilon =\frac{\left(\lambda -\lambda_{res}\right)}{\left(\frac{FWHM}{2}\right)}$ is the normalized detuning quantity, $\lambda_{res}$ is the resonant center wavelength, FWHM is the conjugate half-height full width, and the parameter q is the Fano asymmetric parameter, which determines the symmetry and sharpness of the resonant line shape: when |q|→0, the line shape shows a typical symmetrical depression. When |q|≫1, the line shape approaches the symmetrical Lorentz Peak. The constants A and B represent the amplitude factor and background shift, respectively.

In this design, by precisely optimizing the geometric parameters of the resonator and its distance from the waveguide, we can effectively regulate the coupling strength and discrete state energy, thereby optimizing the q value to the ideal range and exciting sharp Fano resonance spectral lines with extremely high slopes (i.e., extremely sensitive to refractive index changes), laying the foundation for high-sensitivity sensing.

With ε_air_ = 1 defining the relative permittivity of air, the dielectric behavior of silver is characterized by a frequency-dispersive complex permittivity pursuant to the Debye–Drude model:

(2) $$\begin{array}{c} \varepsilon \left(\omega \right)\;=\;\varepsilon_{\text{∞}}+\frac{\varepsilon_{s}-\varepsilon_{\text{∞}}}{1\;+\;i\tau \omega }+\frac{\sigma }{i\omega \varepsilon_{0}} \end{array}$$

The electromagnetic characteristics are mathematically defined by the following constitutive parameters: ω constitutes the optical angular frequency; ω_p_, valued at 1.38 × 10^16^ rad/s, encapsulates the electron plasma frequency of silver; τ, quantified as 7.35 × 10^−15^ s, delineates the electron relaxation time; ε∞, characterizing the high-frequency permittivity limit, assumes the value 3.8344; ε_0_ is the permittivity in vacuum; ε_s_, quantifying the static dielectric response, is determined as −9530.5; and the electrical conductivity σ is established as 1.1486 × 10^7^ S/m [29].

The TM mode equation is given by [30,31]:

(3) $$\tan h\left(k\omega \right)\;=-\frac{2k\alpha_{c}}{k^{2}\;+\;p^{2}\alpha_{c}}$$

The dispersion relation is governed by the wave vector k, with $\alpha_{c}={\left[{k_{0}}^{2}\times (\varepsilon in-\varepsilon m)+k\right]}^{\frac{1}{2}}$ and free-space wave number k_0_ = $\frac{2\pi }{\lambda 0}$. The permittivity ratio P = ε_in_/ε_m_ is defined by the constitutive parameters of the dielectric core (ε_in_) and metallic cladding (ε_m_), corresponding to the aforementioned order.

The finite element method was utilized for modeling and numerical simulations via the COMSOL Multiphysics 5.6 software platform. This software adeptly simulates the interactions between electromagnetic fields and matter in nanophotonic devices by solving coupled partial differential equations, rendering it particularly well-suited for modal analysis and numerical simulations that involve intricate multi-physics couplings.

In the simulation model, the two termini of the MIM waveguide structure are designated as the input and output ports, respectively, with their port types set as numerical. The input port is energized by a laser source distinguished by its high monochromaticity and coherence, and has its wave excitation enabled. Laser energy is introduced into the system through the input port with a power of 1 W/m, while the output port is configured without excitation to facilitate the monitoring of the transmission response. To ensure computational precision and mitigate unphysical reflections, every simulation domain perimeter, with the exception of the ports, is terminated with perfectly matched layers, which efficiently capture outgoing waves. Subsequently, an ultra-fine mesh is applied to the waveguide and TCRSW structures, while other regions are appropriately coarsened to enhance the accuracy of the results while conserving computational resources.

To quantitatively assess the effectiveness of the coupled system, two key parameters, sensitivity (S) and FOM, are employed, defined as follows [32]:

(4) $$S=\frac{\Delta \lambda }{\Delta n}$$

(5) $$\mathrm{FOM}=\frac{S}{\mathrm{FWHM}}$$

where Δn signifies the variation in the refractive index and Δλ is the corresponding resonant wavelength shift.

## 3. Results and Discussion

**Figure 2:**
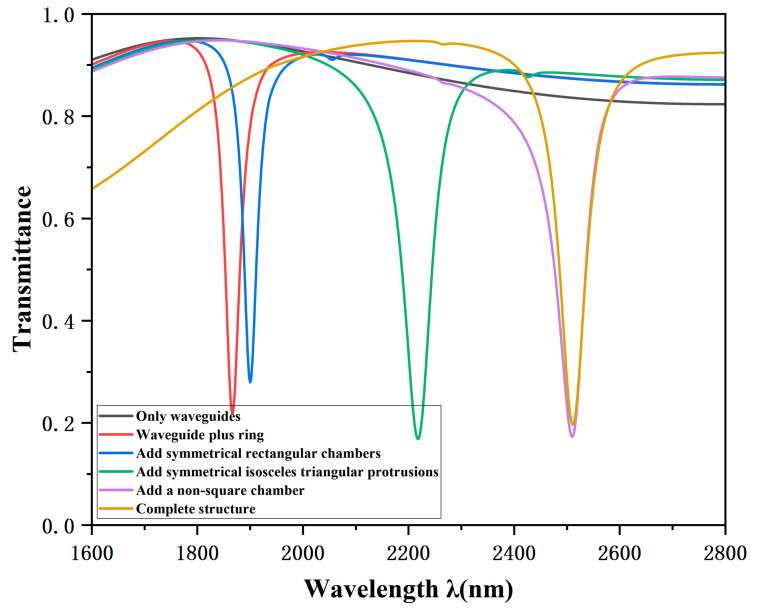
Transmission spectra for the waveguide only (black), waveguide with ring resonator (red), with added symmetric rectangular cavities (blue), with added symmetric isosceles triangular protrusions (green), with an added non-square cavity (purple), and the complete structure (brown).

To elucidate the origin of the Fano resonance, simulations were conducted for individual components and intermediate structures: the standalone MIM waveguide, the structure with symmetric isosceles triangular protrusions, the structure with symmetric rectangular cavities, the structure with a non-square cavity, and the final complete configuration. The corresponding transmission spectra are illustrated in Figure 2. The black curve depicts the isolated MIM waveguide, while the red curve corresponds to the waveguide coupled with the ring resonator, the blue curve depicts the previous structure with the addition of symmetric rectangular cavities, the green curve shows the structure further augmented with symmetric isosceles triangular protrusions, the purple curve includes an additional non-square cavity, and the yellow curve represents the final complete structure incorporating a rectangular stub on the MIM waveguide. All structures share identical geometric parameters: an outer ring radius R of 240 nm, a cavity width ω of 50 nm, a vertex angle θ of 60° for the symmetric equilateral triangular cavities, and a coupling distance g of 15 nm between the waveguide and the TCRSW structure.

An in-depth analysis reveals that the spectral transmittance response of the standalone MIM waveguide (black curve) remains stable and high across the wavelength range of 0.8 to 0.95, approximating a straight line, which can be characterized as a wideband mode. In contrast, the other five curves (red, blue, green, purple, and yellow) each exhibit distinct resonance dips with low transmittance and extremely narrow spectral widths. Their line shapes approximate Lorentzian distributions, indicative of narrowband modes that are directly stimulated by the incident light. Furthermore, the pronounced asymmetry observed in these curves provides clear evidence of successfully excited Fano resonance within the structure. This phenomenon arises from the interference and superposition within the continuum state provided by the MIM waveguide and the discrete states generated by the TCRSW resonator.

**Figure 3:**
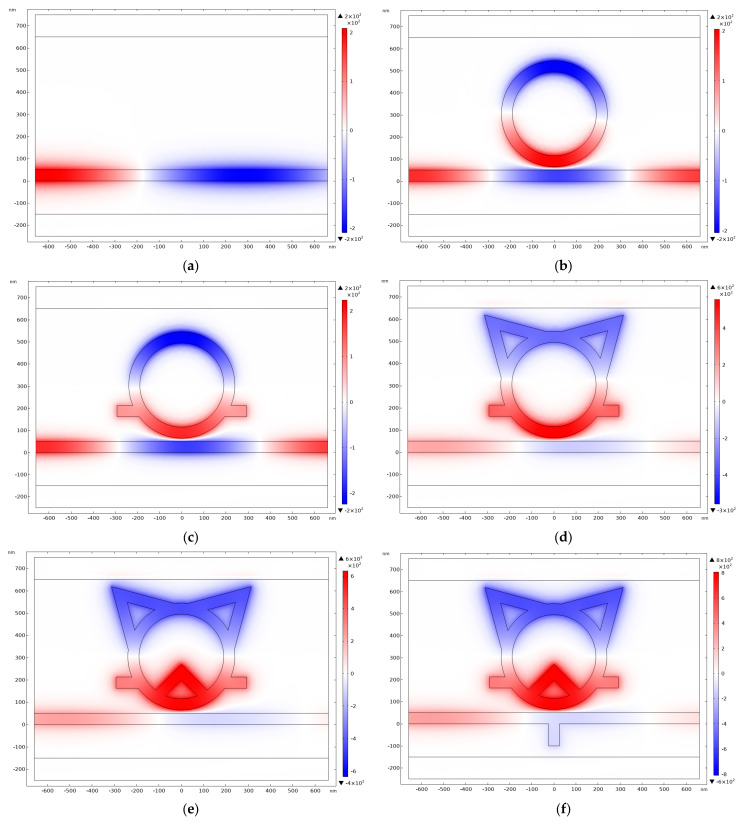
Normalized magnetic field distributions for the structural evolution stages (**a**–**f**).

To delve into the formation mechanism of the TCRSW structure, the normalized distributions of the magnetic field associated with the structural evolution process are plotted in Figure 3, with the sequence progressing from (a) to (f). Each stage in the figure corresponds precisely to the structures characterized by the black, red, blue, green, purple, and yellow curves in Figure 2, respectively.

As illustrated in Figure 2, with the continuous adjustment of structural parameters, the transmission spectrum exhibits a pronounced red shift trend. Concurrently, the electric field distribution is observed to become increasingly concentrated within the resonant cavity as the structure evolves. This enhanced field localization signifies more efficient coupling of Surface Plasmon Polaritons (SPPs) into the resonator interior, with less SPP energy radiating from the output port.

Furthermore, the purple and yellow curves represent the spectral transmittance response for the straight waveguide coupled with the TCRSW and the fully integrated structure (including the rectangular stub), respectively. A comparative analysis indicates that the minimum transmittance of the comprehensive structure is marginally superior to that of the linear waveguide configuration. More importantly, the complete structure exhibits a significantly narrower FWHM. This characteristic significantly enhances the FOM of the resonant structure, indicating that the complete structure with a rectangular barrier added to the waveguide has more pronounced advantages in optical performance.

**Figure 4:**
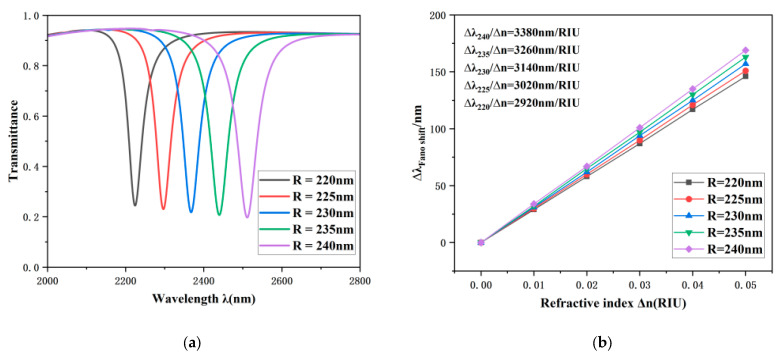
(**a**) Optical transmission properties under different outer radii (*R*) of the TCRSW structure. (**b**) The corresponding sensitivity fitting line as a function of the outer radius.

Subsequently, we investigated the impact of different structural parameters on the transmission performance of the sensing system. The initial parameter settings for the system structure are *R* = 240 nm, *r* = *R* − 50 = 190 nm, *θ* = 60°, *φ* = 90°, *h* = 100 nm, *L* = 100 nm, *g* = 15 nm, and *ω* = 50 nm. To simulate and investigate the effects of varying outer radius R values on the spectral transmission response of the system, experiments were conducted with *R* values set to 220 nm, 225 nm, 230 nm, 235 nm, and 240 nm, in sequence. The experimentally observed responses are illustrated in Figure 4a.

It is clear that as the value of *R* increases, the spectral curve exhibits a distinct red shift. This effect is due to the augmentation of the effective wavelength of the TCRSW structure caused by the enlarged working area of the resonant cavity. In contrast, the alterations in optical transmittance and FWHM are insignificant. As delineated in Figure 4b, as *R* increases, the system sensitivity varies from 2920 nm/RIU to 3380 nm/RIU. These results indicate that the outer radius *R* is a critical factor in the evolution of the structural parameters. In practical applications, this parameter can be specifically tailored in accordance with the sensitivity specifications of the particular device, thereby ensuring performance standards are met while achieving efficient resource utilization and optimal configuration.

**Figure 5:**
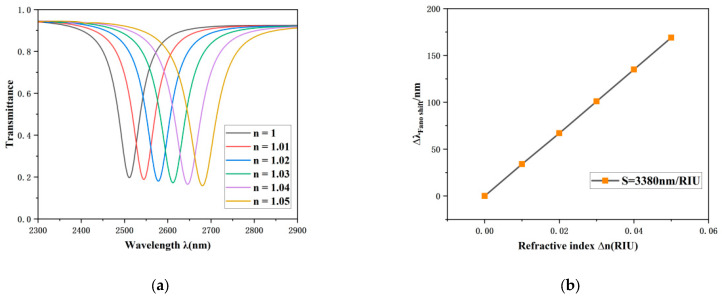
(**a**) Optical transmission properties under different refractive indices. (**b**) Sensitivity fitting curve as a function of the refractive index.

**Figure 6:**
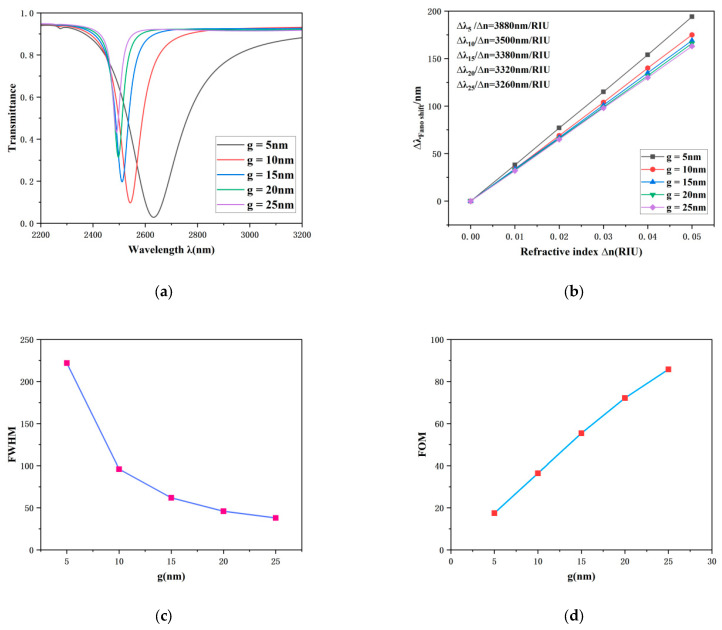
(**a**) Optical transmission properties under different coupling distances (*g*). (**b**) Sensitivity fitting line as a function of the coupling distance. (**c**) FWHM versus the coupling distance. (**d**) Figure of merit (FOM) at different coupling distances.

To further elucidate the impact of the refractive index (*n*) on the transmission characteristics of the sensor system, a comprehensive experiment was conducted wherein *n* was sequentially incremented from a baseline of 1.00 RIU to an upper threshold of 1.05 RIU, with a step size of 0.01 RIU. The transmission spectra related to the various refractive indices are displayed in Figure 5a. The experimental findings reveal that with an increase in the refractive index, the Fano resonance curve exhibits a pronounced red shift phenomenon. Moreover, the sensitivity of the sensing system can be quantitatively assessed through the shift in the resonance dip. As depicted in Figure 6b, the linear regression analysis of the system’s sensitivity indicates a peak attainable sensitivity of 3380 nm/RIU, accompanied by a FOM of 56.33.

Subsequently, the coupling distance (*g*) between the TCRSW structure and the waveguide was investigated, with this study evaluating its multifaceted impact on the performance of the plasmonic sensing system. Figure 6a illustrates the variation in transmission spectra under different coupling distances *g*. As *g* incrementally increases from 5 nm to 25 nm while other structural parameters are kept constant, a significant blue shift in the transmission spectrum is observed, concomitant with a substantial increase in the overall transmittance. This observation elucidates that the coupling efficiency between the SPPs and the TCRSW resonator incrementally decreases with a larger *g*, allowing more energy to radiate from the output port and resulting in a marked reduction in the electric field localization within the resonator.

In practical sensing applications, the selection of an appropriate coupling distance *g* requires a comprehensive consideration of multiple performance metrics. The transmittance should not be excessively high, and FWHM must be maintained within a reasonable range. As shown in Figure 6c, the FWHM exhibits a gradual decreasing trend as *g* increases. The change is more pronounced when *g* < 15 nm, while it stabilizes when *g* > 15 nm. An excessively large FWHM directly leads to a degradation of the FOM, which is detrimental to the system’s resolution.

Regarding sensitivity performance (Figure 6b), across a variation in *g* from 5 nm to 25 nm, the sensitivity fluctuates within a limited range between 3260 nm/RIU and 3880 nm/RIU, suggesting that the coupling distance has a relatively weak influence on sensitivity. In contrast, the FOM undergoes a substantial change: as depicted in Figure 6d, the FOM increases sharply from 17.48 to 85.76 with increasing *g*, underscoring the critical role of *g* in determining the system’s optical quality and sensing precision.

In summary, the selection of the coupling distance *g* necessitates a holistic balance of key indicators including transmittance, sensitivity, FWHM, and FOM. When *g* is less than 15 nm, the FOM decreases drastically, which is unfavorable for high-precision sensing. When *g* exceeds 15 nm, the transmittance increases significantly, the corresponding FWHM change moderates, and the sensitivity remains relatively stable. Consequently, this study identifies the optimal coupling distance as 15 nm. This value ensures high sensitivity while effectively avoiding performance degradation associated with high transmittance and low FOM, thereby achieving an optimal overall sensor configuration.

**Figure 7:**
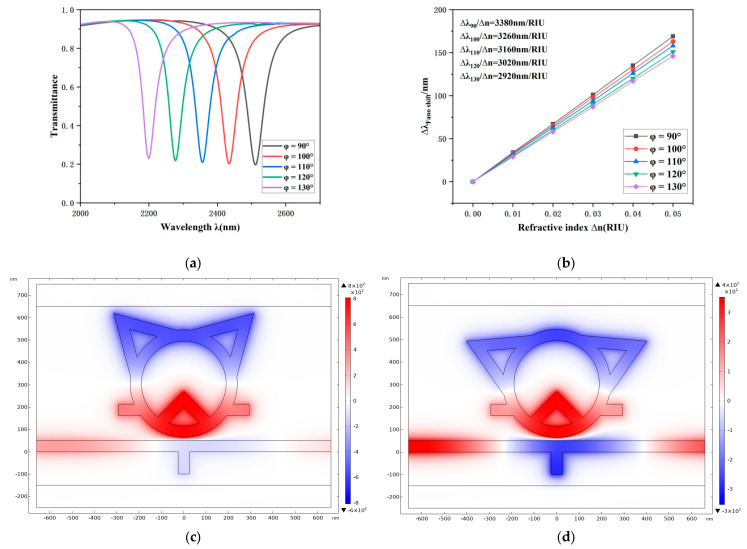
(**a**) Optical transmission properties under different values of the angle *φ*. (**b**) Fitted sensitivity versus azimuthal angle *φ*. (**c**,**d**) Normalized magnetic field distributions of the structure at *φ* = 90° and *φ* = 130°, respectively.

Furthermore, this study systematically examined the influence of the angle (*φ*) between the central axes of the two symmetric triangular resonators relative to the ring center on the system’s transmission characteristics. As illustrated in Figure 7a, the resonance trough in the Fano spectrum exhibits a notable blue shift trend with increasing *φ*. Figure 7c and Figure 7d present the normalized magnetic field distributions related to the TCRSW configuration at points *φ* = 90° and *φ* = 130°, respectively. A pronounced amplification of the electric field within the resonator is observed as *φ* increases, indicating that a smaller apex angle between the dual triangular cavities facilitates stronger concentration of the electric field by the TCRSW structure.

Concurrently, neither the optical transmittance nor the FWHM of the system undergo significant changes, suggesting that variations in *φ* do not markedly affect the spectral linewidth or transmission efficiency. Linear fitting of the system sensitivity (Figure 7b) reveals an improvement from 2920 nm/RIU to 3380 nm/RIU as *φ* increases, demonstrating the positive regulatory role of this parameter on the sensing sensitivity.

These results indicate that the angle *φ* is a critical geometric parameter for optimizing the optical performance of the TCRSW structure. In practical device design and integration, a suitable *φ* value can be determined considering the trade-off between spatial layout constraints and sensitivity requirements. This enables the optimization of spatial utilization efficiency while ensuring sensing accuracy, providing an effective design basis for realizing high-performance plasmonic sensing structures.

**Figure 8:**
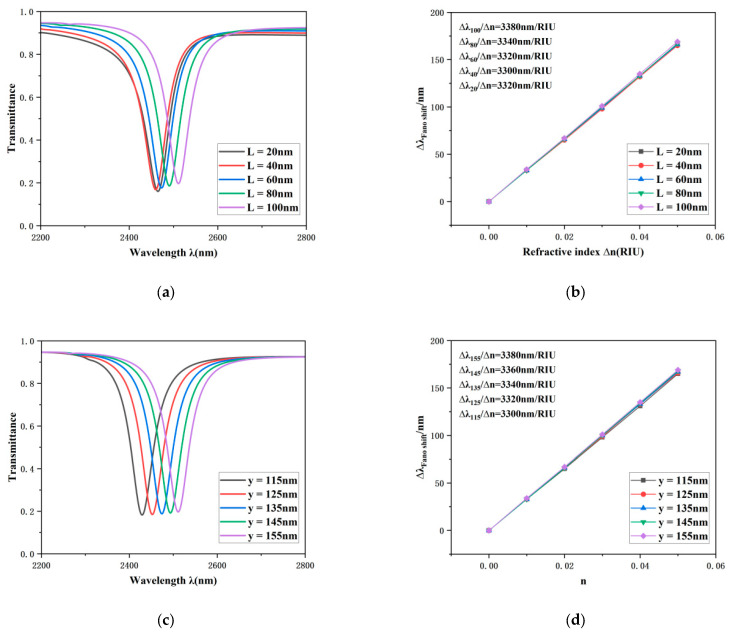
(**a**) Optical transmission properties under different values of the side length *L*. (**b**) Fitted sensitivity parameterized by *L*. (**c**) Optical transmission properties under different values of the leg length *y*. (**d**) Sensitivity fitting curve as a function of *y*.

Finally, the influence of the waist length (*y*) of the external isosceles right-angled triangular cavity and the edge dimension (*L*) of the external equilateral triangular resonators on the performance of the resonant system was investigated. Figure 8a,b illustrate the transmission spectra alongside the corresponding sensitivity fitting curves as the parameter *L* is varied from 20 nm to 100 nm. As shown, the sensitivity transitions from 3320 nm/RIU to 3380 nm/RIU, and the transmission spectrum exhibits a red shift phenomenon. However, no significant changes in sensitivity or FWHM are observed. Similarly, Figure 8c,d present the results when *y* is varied from 115 nm to 155 nm. The sensitivity varies from 3300 nm/RIU to 3380 nm/RIU, accompanied by a red shift in the transmission spectrum, while both sensitivity and FWHM remain largely unaffected.

In summary, within the selected ranges, the parameters *L* and y exhibit limited influence on the transmission characteristics and sensing performance of the system, indicating that they are not sensitive variables in the current structural design. Nonetheless, these parameters remain critical factors in the subsequent fabrication and realization of practical devices.

Finally, we created a performance comparison table between the sensor in this study and other structural sensors, as shown in Table 1.

The sensor structure proposed in this study exhibits significant advantages in multiple aspects, demonstrating not only high optical sensitivity and pronounced Fano resonance wavelength shifts but also excellent potential for practical applications. The structural design is conducive to achieving lightweight, miniaturized, and highly integrated configurations, making it suitable for micro-sensing platforms. From a fabrication perspective, the sensor offers a straightforward and highly reproducible manufacturing process. The primary structure can be readily fabricated through the etching of a 100 nm thick silver layer on a quartz base, thereby substantially minimizing complexity and production costs.

Furthermore, relative to silver and quartz, ethanol displays a refractive index variation range that is two magnitudes greater, making it more suitable for temperature detection applications. The refractive index of ethanol demonstrates a linear covariation with temperature across the inclusive range from its melting point (−114 °C) to boiling point (78 °C). This characteristic endows the TCRSW structure with superior stability and accuracy in temperature sensing. The linear dependence of ethanol’s refractive index on temperature can be expressed by Equation (6).

(6) $$n=1.36048-3.94\times 10^{-4}\times (T-T_{0})$$

where *T* is designated as the ambient temperature under test and T_0_ is established as the reference room temperature of 20 °C. Owing to the liquid state of ethanol across the −114 °C to 78 °C interval, the operational temperature range of the sensor was configured to align with this phase stability range. The structural parameters were established as detailed below: *R* = 240 nm, *ω* = 50 nm, *θ* = 60°, *g* = 15 nm, *L* = 100 nm, *y* = 155 nm, and *φ* = 90°. In this study, the temperature was systematically adjusted to −85 °C, −55 °C, −25 °C, 5 °C, 35 °C, and 65 °C for the purpose of evaluation.

Changes in the ambient temperature induce variations in ethanol’s refractive index. The temperature sensor detects this change via the resulting spectral shift, converting the shift magnitude into temperature information. This approach, based on the spectral shift phenomenon, results in a temperature sensor possessing good precision, stability, and sensitivity. The temperature sensor’s sensitivity can be expressed through the following Equation (7):

(7) $$S_{T}=\frac{∆\lambda }{∆T}$$

where *Δλ* indicates the change in the transmission spectrum profile. Based on the established temperature variation range, *ΔT* is set as 150 °C. Based on the temperature range defined for this experimental investigation, the resulting variation in the refractive index of the fabricated structure, as determined through Equation (6), spans from 1.34275 to 1.40185.

Meanwhile, the definition of temperature sensing FOM is as follows:

(8) $${FOM}_{T}=\frac{S_{T}}{FWHM}$$

Among them, S_T_ represents the sensitivity of the temperature sensor and FWHM is the half-width of the formant peak.

**Figure 9:**
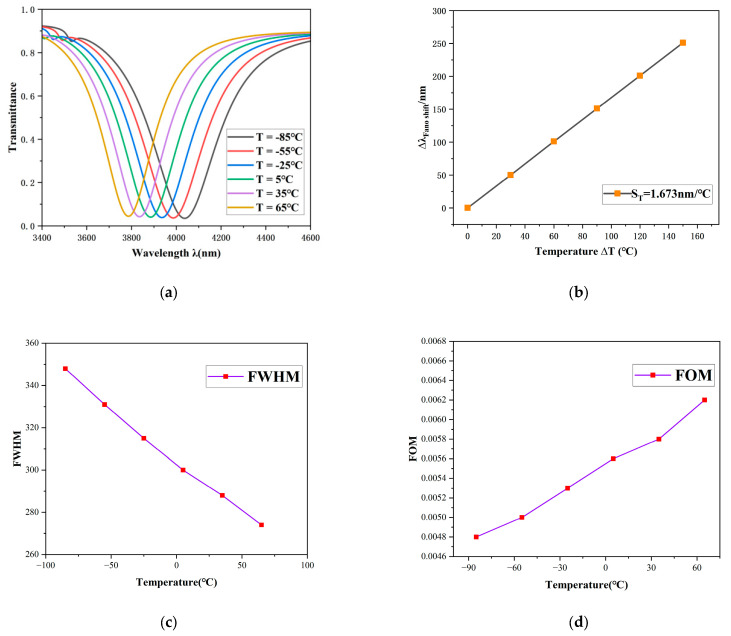
(**a**) Transmission spectra over the temperature range from −85 °C to 65 °C. (**b**) Sensitivity fitting curve as a function of temperature. (**c**) The FWMH corresponding to the sensor system at different temperatures. (**d**) The FOM corresponding to the sensor system at different temperatures.

Figure 9 vividly illustrates the variations in the sensor’s transmission spectrum in response to temperature changes. Figure 9a illustrates a significant blue shift in the transmission spectrum corresponding to an increase in temperature. The transmission dip shifts from 3787 nm to 4038 nm, corresponding to a total spectral shift (*Δλ*) of 251 nm.

Furthermore, as shown in Figure 9b, a strong correlation is observed between the temperature change and the sensor’s optical response. The spectral shift exhibits excellent linear fitting characteristics, providing a reliable basis for the quantitative assessment of the sensor’s sensitivity. The calibrated sensitivity of the sensor reaches 1.673 nm/°C, underscoring its significant potential for high-precision temperature monitoring applications.

Figure 9c,d show the FWHM and FOM corresponding to the sensor system at different temperatures. As the temperature changes from −85 °C to 65 °C, the system’s resonant half-width (FWHM) varies from 348nm to 274nm and the optimum (FOM) changes from 0.0048 to 0.0062. Owing to its remarkable linear response and high sensitivity, this sensor possesses considerable practical value for precision temperature sensing.

**Figure 10:**
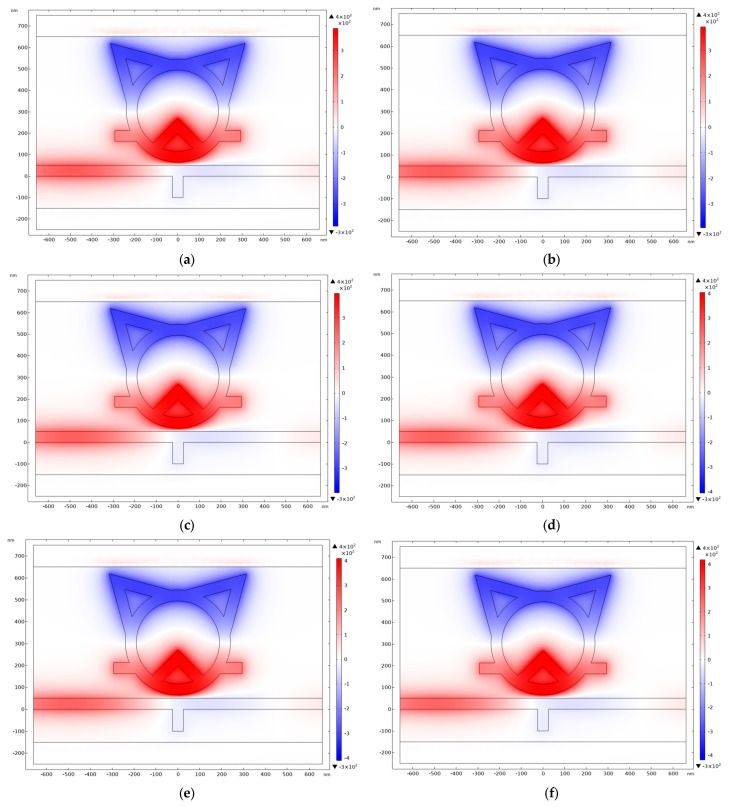
The normalized magnetic field distributions of the system across a temperature range from −85 °C to 65 °C. are presented in Figure (**a**–**f**).

As shown in Figure 10, which presents the normalized magnetic field distribution under temperature variations ranging from −85 °C to 65 °C, it can be observed that the electric field distribution exhibits minimal variation across different temperatures. This indicates that, given the fixed structural parameters of the system, temperature changes do not significantly affect the concentration of the electric field within the structure. Variations in the electric field distribution are primarily governed by changes in the structural parameters of the system. Concurrently, the proposed structural design concept and operational mechanism offer valuable insights and design guidelines for future research in nanophotonic sensors.

In a human physiological fluid environment, sodium ions (Na^+^) and potassium ions (K^+^) constitute the most fundamental and essential electrolyte system. Na^+^ is predominantly distributed in the extracellular fluid and plays a dominant role in maintaining extracellular osmotic pressure and fluid distribution. In contrast, K^+^ is highly concentrated in the intracellular fluid and is a critical ionic species for sustaining normal cellular electrical activity and metabolic functions. Through their coordinated regulation, Na^+^ and K^+^ jointly contribute to the control of body fluid volume, osmotic balance, and acid–base homeostasis [38]. Therefore, real-time monitoring of Na^+^ and K^+^ concentration variations in blood and related bodily fluids is of significant importance in biomedical sensing and clinical diagnostics.

Motivated by this physiological relevance, the present study further investigates the applicability of the proposed structure for ionic concentration sensing. A series of Na^+^ and K^+^ aqueous solutions with different concentrations were introduced into the MIM waveguide and SCAC structure to systematically evaluate the sensing response characteristics. Liquid samples can be rapidly introduced into the sensing region via capillary action, while biological blood samples can be pretreated using blood purification processes prior to detection, thereby reducing contamination and enabling repeated reuse of the sensor.

Under constant temperature conditions, the refractive indices of Na^+^ and K^+^ solutions exhibit concentration-dependent variations with respect to their mass concentrations (mg·dL^−1^), which can be described by the following empirical relationships [39,40]:

(9) $$n_{Na^{+}}=1.3373+1.768\times 10^{-3}\times \frac{C\times k}{393}-5.8\times 10^{-6}\times (\frac{C\times k}{393}{)}^{2}$$

(10) $$n_{K^{+}}=1.3352+1.6167\times 10^{-3}\times \frac{C\times k}{529.8}-4\times 10^{-7}\times (\frac{C\times k}{529.8}{)}^{2}$$

where *C* denotes the concentration (mgd/L) and *k* represents the concentration coefficient, with values of 30 and 50 for Na^+^ and K^+^ solutions, respectively.

The sensitivity can be calculated using the following equation:

(11) $$S_{C}=\frac{\Delta \lambda_{C}}{\Delta C}$$

**Figure 11:**
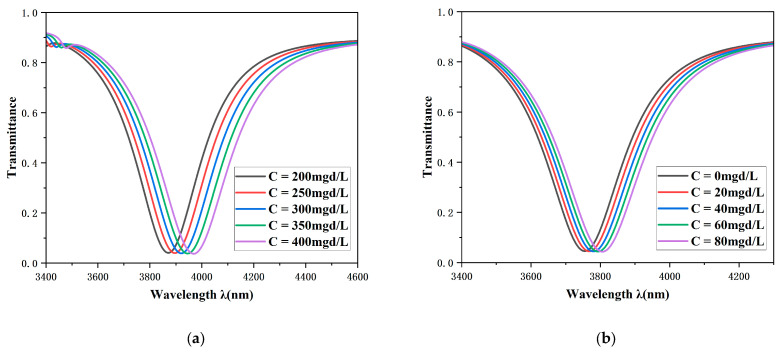
(**a**) Transmission spectra of the Na^+^ solution with concentrations ranging from 200 mg·d/L to 400 mg·d/L; (**b**) transmission spectra of the K^+^ solution with concentrations ranging from 0 mg·d/L to 80 mg·d/L.

Within the normal physiological concentration range, the Na^+^ concentration was set to 200, 250, 300, 350, and 400 mg·d/L, while the K^+^ concentration was configured as 0, 20, 40, 60, and 80 mg·d/L. The corresponding simulation results are presented in Figure 11. As the concentrations of sodium and potassium ion solutions vary within a certain range, a pronounced red shift in the resonance dip in the transmission spectrum can be clearly observed. Further analysis demonstrates that the proposed sensing structure exhibits high sensitivity for the detection of sodium and potassium ion concentrations, with values of 0.49 mg·d/L and 0.6375 mg·d/L, respectively. With the assistance of modern optical detection systems, the spectral response induced by a concentration variation as small as 1 mg·d/L can be accurately resolved. This structure enables continuous and precise monitoring of ion concentrations in human blood, which is of great significance for maintaining physiological homeostasis. In addition, the quantitative determination of sodium and potassium ions in venous serum is a routine yet critical procedure in clinical emergency diagnostics, providing essential information for clinical diagnosis and treatment. Owing to their simple structural design, high operational reliability, and ease of system integration, biosensors offer important technical guidance for the development of surface plasmon resonance-based biosensing technologies.

## 4. Fabrication Feasibility and Tolerance Analysis

### 4.1. Fabrication Feasibility Analysis

The proposed TCRSW-based MIM sensor is fully compatible with state-of-the-art nanofabrication technologies. Owing to its planar and two-dimensional configuration, the entire structure is well suited for fabrication using mature top-down approaches. Specifically, the sensor can be fabricated by depositing a silver film with a thickness of approximately 100 nm onto a quartz substrate, followed by pattern definition using electron-beam lithography (EBL) or focused ion beam (FIB) milling, and subsequent metal etching or lift-off processes. These techniques are widely employed for fabricating MIM plasmonic waveguides and resonator structures with feature sizes well below 50 nm. Importantly, the minimum feature sizes in the proposed design, including the waveguide width (*ω* = 50 nm), the coupling gap (*g* = 15 nm), and the cavity features, fall within the resolution limits routinely achievable by modern EBL and FIB systems.

Although the proposed TCRSW geometry contains multiple embedded cavities and sharp corner features in the idealized simulation model, such geometries are not uncommon in plasmonic device fabrication. In practical fabrication processes, sharp corners are naturally subject to rounding effects due to resist diffusion, proximity effects, and beam scattering. It should be emphasized that the sensing performance of the proposed structure is primarily governed by the overall resonant cavity topology and modal interference mechanism rather than by perfectly sharp vertices. Therefore, moderate corner rounding is not expected to significantly alter the resonance behavior or sensing performance.

Moreover, similar ring resonators, triangular cavities, and composite plasmonic geometries with sharp features have been successfully fabricated and experimentally characterized in numerous previous studies, further demonstrating the feasibility of the proposed design. For example, Zhang et al. experimentally fabricated split-ring resonator/disk nanocavities and observed Fano resonances in transmission spectra in the near-IR region using electron-beam lithography and optical spectroscopy [41]. In addition, Cetin et al. experimentally fabricated asymmetric ring/disk plasmonic nanocavities for biosensing applications and characterized their optical responses on conductive substrates, demonstrating sharp Fano resonance features [42].

### 4.2. Fabrication Tolerance and Error Analysis

In practical fabrication processes, slight deviations in geometric parameters such as the outer radius (*R*), coupling distance (*g*), cavity dimensions (*L* and *y*), and waveguide width (*ω*) are inevitable. However, the parametric studies presented in this work demonstrate that the proposed sensor exhibits robust performance against moderate dimensional variations. Specifically, within the investigated ranges, variations in parameters such as *L* and *y* result in only minor changes in sensitivity and FWHM, indicating that these parameters are low-sensitivity variables. This inherent robustness relaxes fabrication precision requirements and improves device yield.

Among all structural parameters, the coupling distance *g* has the most pronounced influence on the FOM. Nevertheless, the results show that when g varies within a reasonable fabrication tolerance window (±5 nm around the optimized value of 15 nm), the sensor maintains stable sensitivity, while the FWHM and FOM exhibit gradual variations. This behavior indicates that the sensor performance does not rely on an extremely narrow tolerance window, making it resilient to typical nanofabrication uncertainties.

In realistic fabrication conditions, sharp corners are typically rounded, and metal surfaces exhibit finite roughness. These effects may introduce additional plasmonic losses, leading to slight resonance broadening and reduced transmittance contrast. However, since the sensing mechanism is based on relative spectral shifts rather than absolute transmittance values, the sensitivity is expected to remain largely unaffected. Consequently, the sensing functionality and detection accuracy of the device are expected to be preserved under practical fabrication conditions.

Overall, the proposed TCRSW-based MIM sensor demonstrates good tolerance to fabrication-induced imperfections, including dimensional deviations, corner rounding, and surface roughness. The combination of hierarchical resonator design and multi-mode interference results in a structurally robust and practically realizable sensing response. This tolerance, together with compatibility with standard fabrication technologies, supports the feasibility of experimental implementation and practical deployment of the proposed sensor.

### 4.3. Experimental Implementation Route

**Figure 12:**
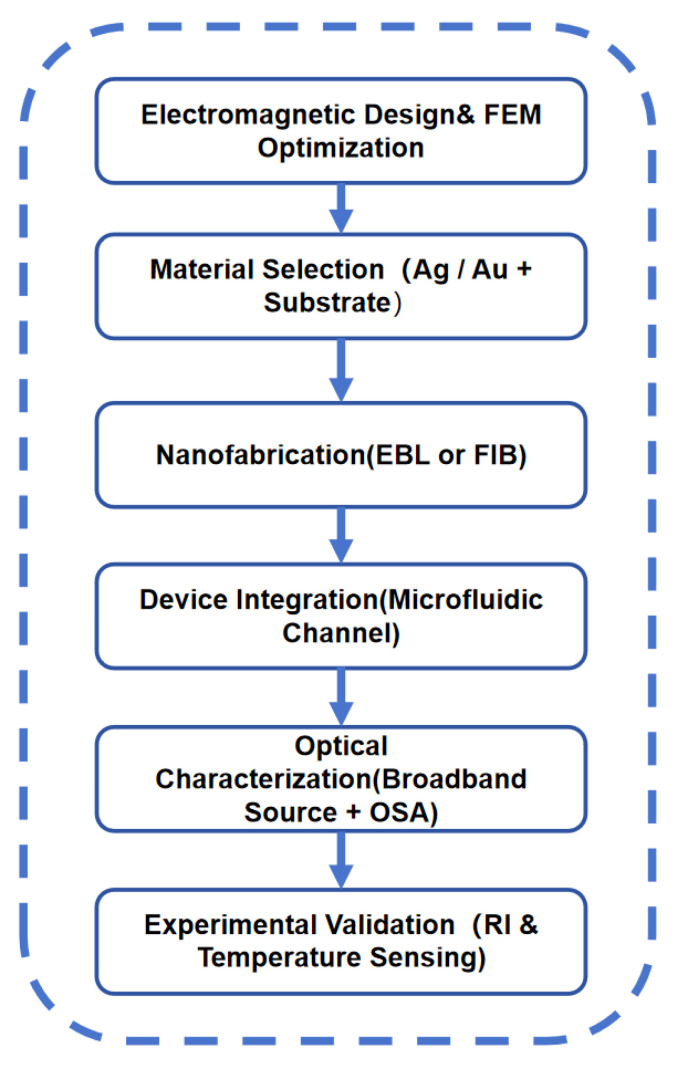
Roadmap toward experimental implementation.

Although the present study is based on numerical simulations, the proposed MIM plasmonic sensor is fully compatible with standard nanofabrication and optical characterization techniques. To address the lack of experimental validation, a clear roadmap toward real implementation is provided in Figure 12. The roadmap includes electromagnetic design and optimization, nanofabrication using electron-beam lithography or focused ion beam techniques, device integration with microfluidic channels, optical characterization, and experimental validation for refractive index and temperature sensing. This roadmap demonstrates the practical feasibility of the proposed design and provides clear guidance for future experimental realization.

## 5. Conclusions

This paper presents a novel refractive index nanosensor that integrates rectangular barriers within a MIM waveguide and a TCRSW resonator. The influence of the sensor’s geometry on its performance metrics was thoroughly assessed through finite element analysis. A series of parametric studies revealed that increases in the values of *R*, *n*, the side dimension of the external equilateral triangular cavities (*L*), and the leg length of the external isosceles right-angled triangular cavity (*y*) consistently resulted in a red shift in the Fano resonance spectrum. Conversely, an augmentation in the value of *g* elicited a pronounced blue shift.

Through comprehensive optimization considering sensitivity, resonance wavelength, FWHM and FOM, the optimal geometric parameters were ascertained as detailed below: *R* = 240 nm, *ω* = 50 nm, *θ* = 60°, *g* = 15 nm, *L* = 100 nm, *y* = 155 nm, and *φ* = 90°. This configuration yielded optimal sensor performance, characterized by a maximum sensitivity of 3380 nm/RIU in conjunction with a FOM of 56.33. Furthermore, the structure exhibited remarkable performance in temperature sensing, registering a sensitivity of 1.673 nm/°C. The application of this structure in biosensing was also investigated. When employed as a concentration sensor for the detection of sodium and potassium ion solutions, the achievable maximum sensitivities reach 0.49 mg·d/L and 0.6375 mg·d/L, respectively.

The proposed sensor architecture demonstrates considerable promise for prospective applications in photonics and nanoscale sensing technologies.

## Figures and Tables

**Table 1:** Comparison of the performance of various sensors.

References	Structure	Sensitivity (nm/RIU)	FOM
[33]	Square Ring Resonator	2473	56.35
[34]	Stub and Ring Hybrid Resonator	1650	117.8
[35]	CSRRC Structure	1114.3	55.71
[36]	L-shaped Structure	1638	21.84
[37]	SERR Structure	1783	27
This Work	TCRSW Structure	3380	56.33

## Data Availability

The original contributions presented in this study are included in the article. Further inquiries can be directed to the corresponding author.
